# Phytase in Pig Diets: Technical and Economic Evaluation

**DOI:** 10.3390/ani16111714

**Published:** 2026-06-03

**Authors:** Danilo de Souza Sanches, Charles Kiefer, Ricardo Carneiro Brumatti, Karina Marcia Ribeiro de Souza Nascimento, Luan Souza dos Santos, Anderson Corassa, Elis Regina de Moraes Garcia, Gislaine da Cunha de Andrade, Giovana Cristina Giannesi

**Affiliations:** 1 Veterinary and Animal Science Department, Federal University of Mato Grosso do Sul, Campo Grande 79070-900, MS, Brazil; rbrumatti@gmail.com (R.C.B.); karina.souza@ufms.br (K.M.R.d.S.N.); santos.luan@ufms.br (L.S.d.S.); andradegislainega@gmail.com (G.d.C.d.A.); 2 Agrarian and Environmental Sciences Institute, Federal University of Mato Grosso, Sinop 78550-000, MT, Brazil; anderson.corassa@ufmt.br; 3 Department of Animal Science, State University of Mato Grosso do Sul, Aquidauana 79200-000, MS, Brazil; ermgarcia@uems.br; 4 Institute of Biology, Federal University of Mato Grosso do Sul, Campo Grande 79074-460, MS, Brazil; giovana.giannesi@ufms.br

**Keywords:** carcass characteristics, exogenous enzyme, nutritional cost, phosphorus reduction, pig farming, meta-analysis, systematic review

## Abstract

Despite the widespread use of phytase in swine diets, there is a need to update the current evidence on its effects on performance, carcass traits, and economic outcomes. This meta-analysis evaluated the impact of phytase supplementation on growth performance, carcass traits, and economic returns in growing–finishing pigs, combining meta-analysis with economic modeling. A systematic review (Web of Science, ScienceDirect, PubMed, and Scopus) revealed 1049 studies, 17 of which met the inclusion criteria (PRISMA). Three treatments were considered: a basal diet (BD), a reduced-P diet without phytase (DRP), and a reduced-P diet with phytase (DRP + P), with effect sizes expressed as weighted mean differences (WMDs). The meta-analysis included performance traits (daily feed intake, DFI; daily weight gain, DWG; feed conversion, FC; and final weight, FW) and carcass traits (carcass weight, CW; carcass yield, CY; backfat thickness, BT; loin eye area, LEA; lean meat percentage, LMP; and lean meat yield, LMY). With respect to BD vs. DRP, P reduction impaired performance and decreased ADG, DFI, and final weight (*p* < 0.001), especially at reductions ≥0.12%. In DRP + P vs. BD, phytase had no significant effects on performance (WMD = 0.008 kg/day) or carcass traits. In DRP + P vs. DRP, phytase improved performance (ADG: +0.068 kg/day; DFI: +0.106 kg/day; FW: +4.630 kg; FC: −0.115; *p* < 0.001), with stronger effects in males and at greater P reductions. In DRP + P vs. DB, carcass traits were not significantly affected, whereas DB vs. DRP reduced LEA (WMD = −1.820, *p* < 0.00), and DRP + P vs. DRP produced subgroup-dependent changes in CW, LEA, and LMY according to sex, phytase source, and diet composition. Economically, phytase increased profit by up to US$ 2.66 (vs. BD) and US$ 5.14 (vs. DRP), remaining advantageous even with enzyme price increases of up to 4×. Overall, phytase supplementation improved performance and economic returns, representing an effective strategy for the development of swine production systems.

## Simple Summary

Phytase is an enzyme that increases phosphorus (P) utilization in swine diets, thereby improving performance, reducing costs, and mitigating environmental impacts. In this study, data from multiple studies were analyzed to compare standard diets, reduced-P diets, and reduced-P diets supplemented with phytase. The results showed that reducing P without enzyme supplementation impaired performance, leading to decreased weight gain and final body weight. Conversely, phytase supplementation in reduced-P diets restores performance and may further improve it. In addition, enzyme supplementation increased production profitability, even under fluctuations in its price. Overall, the use of phytase has proven to be an effective strategy for improving pig production and enhancing system sustainability.

## 1. Introduction

Approximately two-thirds of the P contained in plant-based ingredients is bound to phytic acid molecules, which can chelate positively charged nutrients to form phytate complexes [1]. Nonruminant animals lack endogenous enzymes capable of degrading these molecules, making some P and nutrients inaccessible to the body through the diet [2]. Phytate is a polyanionic molecule that can bind to P, minerals (calcium, iron, zinc, and sodium) [3], amino acids (histidine, lysine, and arginine, among others) [4] and other nutrients, reducing the solubility and bioavailability of proteins, starch, lipids and trace elements [5]. A viable alternative to reduce the negative effects of phytate is the use of phytase. This enzyme catalyzes the hydrolysis of phytate, making this molecule suitable for use in animals [6,7].

Phytase in pig diets improves the digestibility of calcium and P [8], increases the retention and concentration of nitrogen, energy [9,10] and amino acids [11,12], and promotes bone mineralization [13]. These effects are promoted without harming zootechnical performance [14,15] or the carcass characteristics of the animals [16,17], reducing the excretion of P into the environment [18] at a reduced and cost-effective nutritional cost [19,20]. Although there is a vast amount of knowledge about the productive effects of phytase in pigs [21,22,23,24], these benefits also apply to the production of broiler chickens, commercial layers, quails, horses and others [7,25]. However, studies addressing the effects of phytase on economic aspects of production, especially related to the cost of the diet, are lacking.

Therefore, it is necessary to verify whether the enzyme is truly efficient in terms of performance and carcass characteristics, but mainly from the perspective of feed costs. Meta-analysis is a robust and systematic approach that updates the literature by analyzing quantitative data from multiple studies while incorporating qualitative characteristics into the analytical model. This approach increases the accuracy and applicability of the results, providing a more comprehensive and precise understanding of the outcomes and overcoming the limitations inherent to isolated studies [26].

Meta-analytical studies incorporating economic approaches for phytase have been conducted by [27] in broiler breeder flocks and by [28] in broiler chickens. Unlike conventional meta-analyses, [28] proposed a meta-analytical approach using three treatments per study to construct a database. To our knowledge, this methodological strategy has not yet been applied in studies involving pigs.

Thus, this study proposes a meta-analytical approach in which the database is structured using three treatments per study. This structure enabled the identification, from multiple perspectives, of meta-analytic responses to the effects of phytase on growth performance and carcass characteristics. In addition, novel global-scale economic insights were generated, as methodologies that simulate technoeconomic evaluations on the basis of performance and carcass data derived from systematic reviews and meta-analyses are pioneering in research involving growing and finishing pigs.

Therefore, this study aimed to estimate the effect size of phytase supplementation on the performance and carcass characteristics of growing and finishing pigs through a systematic review and meta-analysis. In addition, on the basis of the compiled metadata, an economic modeling approach was developed to assess the technical and economic impact of phytase under different scenarios.

## 2. Materials and Methods

### 2.1. Ethics Declarations

This study relied exclusively on data extracted from published literature. Given that no live animals were used or directly involved at any stage of the research, approval from the Institutional Animal Care and Use Committee was not needed.

### 2.2. Selection of Articles, Eligibility Criteria and Data Collection

To carry out the study, a database was created using indexed scientific articles published between 2016 and 2024. The research question was formulated using the “PICo” strategy, identifying the “Population” as “Pigs”, the “Interest” as “Phytase”, and the “Context” as “Performance” or “Carcass characteristics” [29]. Searches were carried out on the Web of Science, Science Direct, PubMed and Scopus platforms via the keywords “Pigs” AND “Phytase” AND “Growing and finishing”. After the preliminary screening, the search criteria were further refined by incorporating the terms “Performance” and “Carcass characteristics” to narrow down relevant studies. All the articles obtained from each database were exported to EndNote Web (Clarivate Analytics, Philadelphia, PA, USA), which made it possible to organize the bibliographical references and remove duplicate records obtained from the indexing databases. The searches were carried out from September 2022 to May 2025.

**Figure 1:**
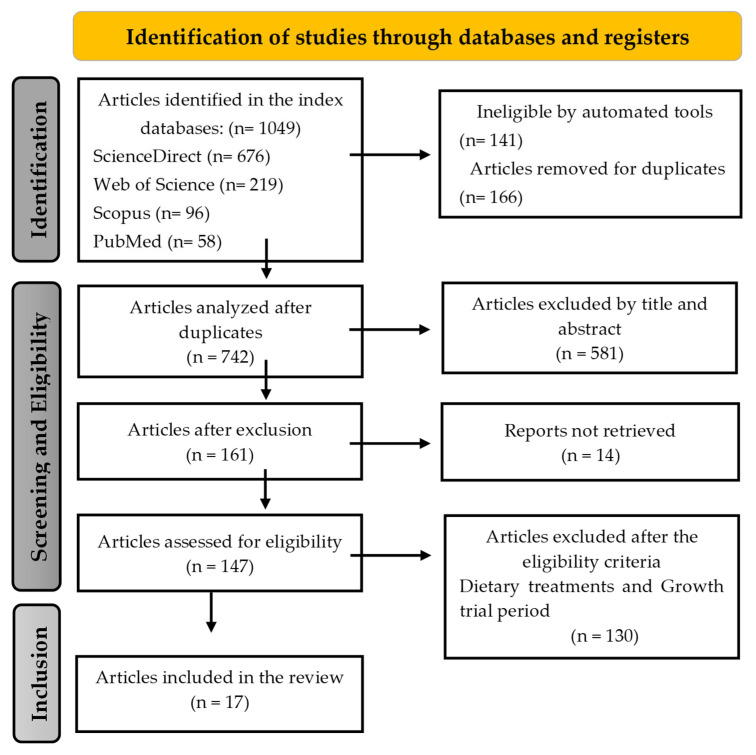
PRISMA flow diagram. Source: Adapted from Page et al. (2020) [30].

The PRISMA (Preferred Reporting Items for Systematic Reviews and Meta-Analyses) flowchart was followed to select the articles [30] (Figure 1). Only articles addressing the performance and carcass characteristics of pigs supplemented with phytase, which met the primary criterion, were considered eligible.

The secondary eligibility criteria were as follows: (a) in vivo studies; (b) studies consisting of at least three diets (a diet formulated according to nutritional requirements and without phytase (BD), a diet formulated with a reduction in digestible P from the nutritional matrix without the inclusion of phytase (DRP) and a diet formulated with a reduction in digestible P from the nutritional matrix containing phytase (DRP + P)); (c) performance studies in the growth and finishing phases; and (d) studies of animal carcass characteristics. In addition, references from the identified publications were also reviewed to identify any additional relevant articles.

### 2.3. Data Systematization and Experimental Diets

The methodology applied for database construction and data coding followed the procedures described by [31]. A total of 1049 publications were found, but only 17 publications were used after the eligibility criteria were applied (Table 1), all of which were used to compose the performance data; however, for the carcass characteristics, only the data published by [14,17,32,33,34,35] were used. The data collected from the articles were tabulated and organized in electronic spreadsheets, and then coding was applied to the data from the growth and finishing phases to facilitate explanation and interpretation. The same coding procedure was used to group the experimental diets.

The number of animals (replicates per treatment and pigs per replicate), sex, origin and phytase level (FTU/kg), reduction in digestible P, and initial and final body weights were recorded. Means and standard deviations for all performance and carcass variables were extracted for the BD, DRP, and DRP + P treatments. These data were subsequently used to compute the weighted mean difference (WMD), heterogeneity, and publication bias for each dependent variable.

For the technical economic analysis, diets based on corn and soybean meal were formulated according to the nutritional requirements of castrated male pigs of high genetic potential, with medium to superior performance in the 30–50 kg, 50–70 kg, 70–100 kg and 100–120 kg phases, according to the nutritional recommendations of Rostagno et al. (2017) [45]. DB was formulated with adequate P, whereas DRP and DRP+F were calculated with a −0.12% reduction in digestible P in all phases. This percentage was calculated on the basis of the P reductions applied in the eligible studies.

### 2.4. Quotation of Feed Ingredients

The price of the ingredients was taken from the database of the Center for Advanced Studies in Applied Economics (CEPEA/Esalq/USP) and the Chicago Stock Exchange through the average prices of ingredients from 2020 to 2025, except for bicalcium phosphate, limestone, premix, inert, amino acids and phytase, which were quoted from different national suppliers. The values quoted in reais were converted into dollars (US$ 1.00 = R$ 5.25) according to the average value obtained from the central bank’s five-year history. In addition, the model incorporated a PHY (Quantum Blue®, AB Vista, Marlborough, Wiltshire, UK) dose of 500 FTU/kg and the live pig market price provided by the local swine industry. FTU refers to the number of phytase units and is defined as the amount of enzyme required to release 1 μmol of inorganic P per minute from 0.0051 mol/L sodium phytate at pH 5.5 and 37 °C [46].

The values of the ingredients (kg) quoted were corn = US$ 0.188, soybean meal = US$ 0.415, soybean oil = US$ 0.674, bicalcium phosphate = US$ 0.776, calcitic limestone = US$ 0.023, L-lysine = US$ 3.233, DL-methionine = US$ 7.759, L-threonine = US$ 5.990, L-tryptophan = US$ 16.784, salt = US$ 0.508, premix = US$ 10.906, inert = US$ 0.041, and phytase = US$ 17.142. The trading price for live pigs was US$1.376/kg.

### 2.5. Growth Performance

On the basis of the formulations made, nutritional costs were generated for each of the phases, and their application to the performance and carcass characteristic results obtained in each study was evaluated. For performance, initial weight (IW), daily feed intake (DFI), digestible P consumption (PC), daily weight gain (DWG), feed conversion (FC) and final weight (FW) were evaluated. To obtain the PC, the following calculation was used: PC,g = (DFI total phase × average digestible P requirement (%) of phases)/100.

### 2.6. Assessment of Carcass Traits

The carcass characteristics analyzed were carcass weight (CW/kg), carcass yield (CY/%), bacon thickness (BT/mm), loin eye area (LEA/cm^2^), lean meat percentage (LMP/%), lean meat yield (LMY/kg) and bonus index (BI). The bonus index was estimated via the equation suggested by Guidoni (2000): IB = 23.6 + 0.286 × hot carcass weight + percentage of meat in the cold carcass [47].

The variables for feed costs were RNP revenue (nonsubsidized pigs), BF revenue (subsidized pigs), feed cost/pig produced, profit, gross margin (GM), feed cost/kg of pig, feed cost/ton of pig, and feed cost variation. The variation in food cost was calculated from the DRP and DRP + P in relation to the control diet.

The revenue equation for the bonus system (IB) proposed by Guidoni (2000) was as follows: IB Revenue = R$/PV × ((PCarc/%Rend) × (23.6 + (0.286 × PCarc) + % CarnM), where R$/PV = price paid to the producer per kilo of live pig; PCarc = carcass weight; %Rend = percentage of carcass yield; and %CarnM = percentage of lean meat in the carcass [47].

### 2.7. Calculations and Statistical Analysis

The performance and carcass data were subjected to meta-analysis, whereas the economic data were analyzed by ANOVA (F test) and, when significant, by Tukey’s test at the 5% significance level.

#### 2.7.1. Effect Size

The effect size for each performance and carcass variable, in comparisons between DB and DRP, DRP + P and DB, and DRP + P and DRP, was expressed as the weighted mean difference (WMD) with 95% confidence intervals. The weight assigned to each study was inversely proportional to its variance, giving greater weight to more precise estimates [48]. The normality of the residuals was assessed using the Shapiro–Wilk test, and the significance of the WMDs was evaluated using the Z test.

#### 2.7.2. Heterogeneity, Meta-Regression and Subgroup Analyses

Heterogeneity between studies was assessed using a random-effects model (restricted maximum likelihood). Its significance was tested using the chi-square test, and its magnitude was quantified using the I^2^ statistic. I^2^ values of <25%, 25–50%, 50–75%, and >75% were interpreted as indicating low, moderate, high, and very high heterogeneity, respectively [49].

Following Vieira et al. (2017), even when heterogeneity was not significant for a given variable, meta-regression and subgroup analyses were performed to explore potential sources of variability [50]. Meta-regression was used as a screening tool to identify relationships between moderators and effect sizes. However, even in the absence of statistical significance, additional subgroup analyses were performed to improve model interpretation.

Subgroups were defined according to the phytase source (*A. oryzae*, *T. reesei*, and others), dietary phytase dose (≤1000 or ≥1001 FTU/kg), reduction in digestible P (≤0.11 or ≥0.12%), basal diet type (corn–soybean or alternative ingredients), and sex (barrows-castrated males, males-as reported in the original studies, or mixed-female and male).

#### 2.7.3. Sensitivity and Prediction Interval Analyses

Given the high heterogeneity observed in some meta-analytic estimates, complementary analyses were performed using the 95% prediction interval (95% PI) [51] and leave-one-out sensitivity analysis [52]. The 95% PI was estimated for each comparison to account for residual between-study heterogeneity and to estimate the expected range of true effects in future independent studies. In the leave-one-out analysis, each study was sequentially excluded, and the meta-analytic model was re-estimated to evaluate changes in the pooled effect size and prediction interval bounds; substantial changes were interpreted as evidence of potential study influence.

#### 2.7.4. Publication Bias

Publication bias was assessed using Egger’s test and considered significant at *p* < 0.05 [53]. Effect size, heterogeneity, and publication bias analyses were performed using the metafor package (version 4.8-0; Wolfgang Viechtbauer, Maastricht University, Maastricht, The Netherlands) in R (version 4.5.1; R Foundation for Statistical Computing, Vienna, Austria).

### 2.8. Feed Cost and Economic Indicators

The performance data of the animals fed the DB, DRP, and DRP + P diets were incorporated into the equations, and the differences among the outcomes were used to estimate the economic impact. The feed costs (US$/kg) were defined for each production phase corresponding to DB, DRP, and DRP + P. The baseline phytase price adopted in the economic analysis was obtained from the average quotation of national suppliers, representing current commercial market conditions. The phosphorus reduction levels applied in the DRP and DRP + P diets were established on the basis of the mean phosphorus reduction values adopted among the eligible studies included in the meta-analysis, aiming to reflect practical nutritional adjustments supported by scientific evidence. The values adopted were as follows: Growing I (0.325, 0.316, 0.317), Growing II (0.303, 0.295, 0.296), Finishing I (0.287, 0.281, 0.282), and Finishing II (0.276, 0.270, 0.272).

The feed costs of the diets per phase were obtained from the following equations: cost of the diet per phase (CDP) = cost/kg of the diet in the phase × DFI of the phase; total diet cost (TDC): sum of feed costs for all phases; feed cost per pig produced (cost/head) = TDC/DWG; food cost variation DRP = ((cost/head DRP × 100)/BD cost/head) − 100; food cost variation DRP + P = ((cost/head DRP + P × 100)/BD cost/head) − 100; feed cost per kg of pig (cost/kg) = feed cost per kg of pig/FW; feed cost per ton of pigs = cost/kg × 1000; revenue = kg pig price (US$) × FW; profit = revenue − cost/kg; gross margin (GM) = profit/revenue.

Sensitivity analysis was used to evaluate the impact of increases in the price of phytase on economic indicators by considering the current phytase price (2× baseline) and simulated scenarios of 3× and 4× the baseline phytase cost, which represented potential market price fluctuations [54].

## 3. Results

### 3.1. Database Characterization

The seventeen studies included in the meta-analysis included a total population of 3239 growing–finishing pigs, distributed as Mixed (n = 7), Male (n = 5), and Barrows (n = 5). Most experimental diets were formulated with corn and soybean meal (n = 7), followed by diets containing alternative ingredients (n = 10). Phytase inclusion levels ranged from 250 to 4500 FTU/kg, and the source varied between *A. oryzae* (n = 6), *T. reesei* (n = 3), and other sources (n = 6). The average reduction in digestible P was 0.11%. The average initial and final weights of the pigs were 27.73 ± 6.96 kg and 112.11 ± 10.30 kg, respectively. The average experimental period was 92 days.

The variables digestible P intake and the carcass bonus index were not included in the meta-analyses because these values were calculated by the authors on the basis of performance variables and carcass characteristics reported in the studies rather than being directly extracted from the original publications. Therefore, descriptive mean values are presented to facilitate the interpretation of the meta-analytical results. The mean values for P intake (%) were 0.27, 0.15, and 0.15, whereas those for P intake (g/day) were 6.75, 3.69, and 3.73 for the BD, DRP, and DRP + PHY groups, respectively. The carcass bonus index values were 105.33, 104.91, and 105.19 for BD, DRP, and DRP + PHY, respectively.

### 3.2. Meta-Analysis of Performance

No outliers were detected, and the studentized residuals of the WMDs followed a normal distribution for all the variables. The overall effects of phytase supplementation on performance across groups are summarized in Table 2, whereas the results of the moderator and subgroup analyses are presented in Table 3 and Table 4, respectively.

In the comparison between DB and DRP, the DB group had increases of 0.06 kg/day in the DWG (*p* < 0.001), 0.09 kg/day in the DFI (*p* < 0.001), and 4.62 kg in the FW (*p* < 0.001), with no effect on the FC (*p* = 0.108). Significant heterogeneity was detected for DWG (*p* < 0.001, I^2^ = 89%), DFI (*p* = 0.014, I^2^ = 74%), FC (*p* < 0.001, I^2^ = 88%), and FW (*p* < 0.001, I^2^ = 12%), suggesting the influence of moderators. Meta-regression revealed that P reduction (*p* < 0.001) was a significant factor affecting FW. Subgroup analysis confirmed that compared with DRP, DB increased FW, with a P reduction ≥ 0.12% (WMD = 6.779 kg, *p* < 0.001).

In the DRP + P vs. DB comparison, phytase did not affect DWG (*p* = 0.374), DFI (*p* = 0.433), FC (*p* = 0.515), or FW (*p* = 0.077). However, significant heterogeneity was observed for DWG (*p* < 0.001, I^2^ = 73%), DFI (*p* = 0.014, I^2^ = 40%), FC (*p* < 0.001, I^2^ = 91%), and FW (*p* < 0.001, I^2^ = 2%), suggesting the influence of moderators. Meta-regression revealed sex (*p* = 0.039) as a significant factor affecting the DFI and P reduction (*p* = 0.008) as a factor affecting FC. However, subgroup analyses for sex and P reduction did not reveal significant sources of variation between the evaluated groups (*p* > 0.05).

In the DRP + P vs. DRP comparison, phytase supplementation increased the DWG by 0.07 kg/day (*p* < 0.001), the DFI by 0.10 kg/day (*p* < 0.001), and the FW by 4.63 kg (*p* < 0.001) and improved the FC by 0.11 (*p* < 0.001). Significant heterogeneity was observed for DWG (*p* < 0.001, I^2^ = 93%), DFI (*p* < 0.001, I^2^ = 84%), FC (*p* < 0.001, I^2^ = 93%), and FW (*p* < 0.001, I^2^ = 10%), suggesting the influence of moderators. Meta-regression revealed sex (*p* = 0.019) as a significant factor affecting FC and reduced P (*p* < 0.001) as a significant factor affecting FW. Subgroup analysis revealed that DRP + P improved FC in males (WMD = −0.313 kg/kg, *p* < 0.001) and increased FW when the reduction in dietary P was ≥0.12% (WMD = 4.873 kg, *p* < 0.001).

Publication bias, assessed using Egger’s test, was not significant for most of the variables evaluated (*p* > 0.05). However, significant asymmetry was detected for FC and FW (*p* < 0.05) across all comparisons.

#### Prediction Interval and Sensitivity Analyses

Additional analyses indicated high robustness of the meta-analytic estimates for performance, with no disproportionate influence of individual studies on the pooled effects. Leave-one-out analysis revealed only minor fluctuations in the pooled estimates and prediction intervals, confirming the strong structural stability of the models.

For DWG, the 95% prediction intervals were wide across all comparisons (DB vs. DRP: −0.0990 to 0.2522; DRP + P vs. DB: −0.1062 to 0.0870; DRP + P vs. DRP: −0.3499 to 0.1777), indicating substantial residual heterogeneity, although leave-one-out analysis confirmed high analytical consistency. A similar pattern was observed for the DFI, with wide 95% prediction intervals (DB vs. DRP: −0.1315 to 0.2291; DRP + P vs. DB: −0.0645 to 0.0518; DRP + P vs. DRP: −0.2824 to 0.1756), suggesting moderate-to-high residual heterogeneity while stable pooled estimates were maintained. With respect to the FC, the 95% prediction intervals also remained wide (DB vs. DRP: −0.1595 to 0.1077; DRP + P vs. DB: −0.1115 to 0.1556; DRP + P vs. DRP: −0.0861 to 0.1820), indicating that the residual variability of the study did not compromise the analytical robustness of the models.

In contrast, FW had relatively narrower 95% prediction intervals (DB vs. DRP: −0.0427 to 0.1264; DRP + P vs. DB: −0.0205 to 0.0067; DRP + P vs. DRP: −0.1089 to 0.0275), indicating lower residual heterogeneity, greater predictive consistency, and high structural stability. Overall, these findings indicate that the observed performance effects were supported by the available evidence rather than driven by isolated studies.

### 3.3. Meta-Analysis of Carcass Characteristics

No outliers were detected, and the studentized residuals of the WMDs followed a normal distribution for all the variables. The overall effects of phytase supplementation on carcass characteristics are summarized in Table 5, and the results of the moderator tests and subgroup analyses are presented in Table 6 and Table 7, Table 8 and Table 9, respectively.

In the DB vs. DRP comparison, DB reduced the LEA by 1.82 cm^2^ (*p* < 0.001), with no effects on CW (*p* = 0.143), CY (*p* = 0.906), BT (*p* = 0.701), LMP (*p* = 0.617), or LMY (*p* = 0.277). Significant heterogeneity was detected for CW (*p* < 0.001, I^2^ = 80%), CY (*p* < 0.001, I^2^ = 65%), BT (*p* < 0.001, I^2^ = 80%), LMP (*p* = 0.010, I^2^ = 60%), and LMY (*p* < 0.001, I^2^ = 98%), suggesting the influence of moderators. Meta-regression revealed P reduction, diet type, and sex (*p* < 0.001) as significant factors affecting CW, and diet type and sex (*p* < 0.001) as significant factors affecting BT, LEA, and LMY. Subgroup analysis revealed that DB reduced the LEA in barrows (WMD = −3.334 cm^2^; *p* < 0.001) and in diets formulated with corn–soybean (WMD = −2.533 cm^2^; *p* = 0.005) and increased the LMY in males (WMD = 0.970 kg; *p* = 0.041).

In the DRP + P vs. DB comparison, phytase had no significant effect on CW (*p* = 0.971), CY (*p* = 0.902), BT (*p* = 0.573), LEA (*p* = 0.110), LMP (*p* = 0.523), or LMY (*p* = 0.201). However, significant heterogeneity was observed for CW (*p* = 0.038; I^2^ = 29%), BT (*p* < 0.001; I^2^ = 68%), and LMP (*p* < 0.001; I^2^ = 60%), indicating the influence of qualitative moderators. Meta-regression analysis revealed that the phytase level (*p* = 0.005) was a factor affecting CW, diet (*p* = 0.001) affected CY, all moderators (*p* < 0.001) affected LMP, and P reduction (*p* = 0.046) affected LMY. No subgroup was significant for carcass characteristics (*p* > 0.05).

In the DRP + P vs. DRP comparison, phytase did not affect CW (*p* = 0.178), CY (*p* = 0.908), BT (*p* = 0.390), LEA (*p* = 0.610), LMP (*p* = 0.951), or LMY (*p* = 0.264). Significant heterogeneity was observed for CW (*p* < 0.001, I^2^ = 72%), LEA (*p* = 0.016, I^2^ = 38%), and LMY (*p* < 0.001, I^2^ = 98%), indicating the influence of qualitative moderators. Meta-regression revealed that all the moderators (*p* < 0.001) significantly affected CW, LEA, and LMY. In addition, the phytase source (*p* = 0.002) was identified as a significant factor affecting CY. However, no moderators were identified for BT or LMP (*p* > 0.05). Subgroup analysis revealed that DRP + P increased CW in males (WMD = 2.666 kg, *p* = 0.011), with phytase derived from *A. oryzae* (WMD = 2.057 kg, *p* < 0.001) or *T. reesei* (WMD = 2.267 kg, *p* = 0.012), at inclusion levels ≤ 1000 FTU/kg (WMD = 1.990 kg, *p* < 0.001), and in diets based on alternative ingredients (WMD = 2.234 kg, *p* = 0.012).

For LEA, subgroup analysis revealed that DRP + P reduced the LEA in barrows (WMD = −2.063 cm^2^, *p* < 0.001), with phytase from *A. oryzae* (WMD = −2.234 cm^2^, *p* < 0.001), at inclusion levels ≤ 1000 FTU/kg (WMD = −2.660 cm^2^, *p* < 0.001), in corn–soybean meal–based diets (WMD = −2.050 cm^2^, *p* < 0.001), and with a P reduction ≤ 0.11% (WMD = −2.063 cm^2^, *p* < 0.001). No significant moderators were identified for CY (*p* > 0.05). An increase in LMY was observed for DRP + P in male pigs (WMD = 1.670, *p* < 0.001), with phytase from *A. oryzae* (WMD = 1.089, *p* = 0.001) or *T. reesei* (WMD = 1.670, *p* < 0.001), at inclusion levels ≤ 1000 FTU/kg (WMD = 1.143, *p* = 0.004).

Publication bias, assessed using Egger’s test, was not significant for most of the variables evaluated (*p* > 0.05). However, significant asymmetry was detected for CY (*p* < 0.05) in the DRP + P vs. DB comparison and for LEA and LMA (*p* < 0.05) in the DB vs. DRP and DRP + P vs. DB comparisons, respectively.

#### Prediction Interval and Sensitivity Analyses

Sensitivity analyses revealed heterogeneous responses across carcass traits. Overall, CY, LEA, and LMP exhibited greater analytical stability and lower residual heterogeneity, whereas CW and, particularly, LMY showed greater residual heterogeneity and greater sensitivity to the influence of individual studies.

For CW, the 95% prediction intervals were wide across all three comparisons (DB vs. DRP: −0.1401 to 0.2514; DRP + P vs. DB: −0.0516 to 0.0629; DRP + P vs. DRP: −0.2016 to 0.1175), indicating moderate-to-high residual heterogeneity. Although leave-one-out analysis confirmed good structural stability, [34] exerted a relevant influence on pooled estimates and residual heterogeneity. In contrast, CY showed narrow 95% prediction intervals (DB vs. DRP: −0.0359 to 0.0379; DRP + P vs. DB: −0.0073 to 0.0027; DRP + P vs. DRP: −0.0048 to 0.0052), indicating low residual heterogeneity, high predictive consistency, and no evidence of influential studies.

For BT, the 95% prediction intervals were relatively wide for DB vs. DRP (−0.1643 to 0.1878) and DRP + P vs. DB (−0.1638 to 0.2137) but narrow for DRP + P vs. DRP (−0.0128 to 0.0309), indicating moderate residual heterogeneity with high analytical robustness. LEA showed relatively narrow 95% prediction intervals (DB vs. DRP: −0.0727 to 0.0110; DRP + P vs. DB: −0.0504 to 0.0011; DRP + P vs. DRP: −0.0555 to 0.0710), suggesting low residual heterogeneity, greater uniformity in response direction, and high structural stability. Similarly, LMP showed consistently narrow 95% prediction intervals (DB vs. DRP: −0.0418 to 0.0327; DRP + P vs. DB: −0.0483 to 0.0348; DRP + P vs. DRP: −0.0061 to 0.0056), indicating high experimental uniformity and strong structural robustness.

Contrasting patterns were observed for LMY, with relatively narrow 95% prediction intervals for DRP + P vs. DB (−0.0437 to 0.0232) and extremely wide intervals for DB vs. DRP (−1.6154 to 2.4720) and DRP + P vs. DRP (−2.4667 to 1.5905), indicating high residual heterogeneity. This heterogeneity was strongly influenced by [34], whose exclusion reduced pooled estimates and narrowed the prediction intervals.

### 3.4. Feed Cost and Economic Indicators

The diets did not affect (*p* > 0.05) revenue from bonified pigs (BF), nutritional cost per head, profit, gross margin, feed cost per kg, feed cost per ton, or cost variation. However, a significant effect (*p* < 0.05) was observed for revenue from nonbonified pigs (RNP) (Table 10). Compared with the BD and DRP + P treatments, the DRP diet reduced RNP by 5.716 and 3.338 US$, respectively.

The sensitivity analysis demonstrated a positive economic impact under all the evaluated conditions. Even when the commercial price of phytase increased to four times the baseline value. On average, compared with the BD treatment, phytase supplementation in diets with reduced phosphorus content, considering the baseline market price as well as 2×, 3×, and 4× increases in phytase cost, improved profit by US$ 2.663, US$ 2.454, US$ 2.069, and US$ 1.727, respectively, and by US$ 5.143, US$ 4.934, US$ 4.549, and US$ 4.207, respectively. In all the simulated pricing scenarios, phytase supplementation remained economically advantageous.

## 4. Discussion

### 4.1. Performance

As expected, compared with the DRP treatment, the DB and DRP + P treatments improved the performance, confirming that compared with the DRP treatment, the phytase supplementation treatment increased the DWG by 0.07 kg/day and the FW by 4.63 kg, reduced the FC by 0.11 kg, and slightly increased the DFI by 0.10 kg/day. These results confirm the role of phytase in improving P utilization and animal performance. The combined analysis improved precision and revealed consistent effects not detected in most individual studies, as expected in meta-analyses [50].

Diets that meet nutritional requirements are essential for realizing the genetic potential of animals [55]. Reducing dietary P without strategies to maintain its availability impairs swine performance because of reduced P absorption efficiency or increased bone mobilization [56]. Furthermore, pigs are unable to efficiently hydrolyze phytate compounds present in plant-based feedstuffs because they do not produce sufficient endogenous phytase capable of breaking phytate bonds, which explains the inclusion of phytase in their diets [57].

These findings support those of Dersjant-Li et al. (2017), who reported increased DWG in growing–finishing pigs with 1000 FTU/kg of phytase [14]. Similarly, Grela et al. (2020) reported that 500 and 1000 FTU/kg improved DWG and FC, respectively, without affecting the DFI. In contrast, Buzek et al. (2023) reported no effect on DWG and DFI but observed improved FC, as did Kasprowicz-Potocka et al. (2020), who reported no effect on DWG in diets based on alternative ingredients with reduced Ca and P [39,44,58].

In the present study, the performance of the pigs fed the DRP diet decreased; however, compared with those fed the DB diet, all the performance parameters improved in response to phytase supplementation. These results may be attributed to the ability of phytase to mitigate the antinutritional effects of phytate, thereby enhancing the digestibility of P, calcium, amino acids, and energy, which consequently improves DWG, FC, and FI [3].

Phytase (myo-inositol hexakisphosphate phosphohydrolase; IP6) acts on the phytate molecule through sequential dephosphorylation, generating lower levels of inositol phosphates (IP5, IP4, IP3, and potentially IP2 and IP1) by removing phosphate groups from myo-inositol esters and releasing orthophosphate [59]. This process also liberates P, amino acids, minerals, and other nutrients bound to phytic acid, which would otherwise be unavailable for absorption in the intestinal lumen [60].

Despite the reduction in dietary P, phytase supplementation improved weight gain, demonstrating its positive effect on pig performance. Jlali et al. (2024) demonstrated that reductions in digestible P of 0.12–0.15 percentage points in weaned piglets and 0.10–0.14 in growing–finishing pigs impair performance, reduce P and calcium digestibility and retention, and compromise bone mineralization [13]. However, supplementation with 500 FTU/kg phytase improved these parameters, partially mitigating the adverse effects of reduced dietary digestible P. Babatunde and Adeola (2022) reported that compared with unsupplemented low-P diets, the inclusion of phytase in low-P diets improves the feed efficiency of pigs by 11% [41].

In the DB vs. DRP and DRP + P vs. DRP comparisons, the DFI was reduced by 0.098 and 0.106 kg/day, respectively, in the pigs fed the DRP diet. These results suggest limitations in P intake and availability, as animals fed the DRP diet consumed lower amounts of this nutrient because of the reduced level of digestible P in the diet. Low P intake may impair essential metabolic processes, such as bone mineralization and energy metabolism, thereby negatively affecting performance [61].

In addition, P deficiency may trigger adaptive physiological responses, such as increased bone resorption and changes in appetite, which may have contributed to reduced food intake and, consequently, lower DWG and FW. These findings indicate that a reduction in dietary P, when not adequately compensated, limits animal performance.

According to Bunzen et al. (2012), variations in the DFI indicate that pigs are sensitive to dietary P levels, as both deficiency and excess reduce food intake [62]. P is an essential mineral for the growth and development of pigs, as it contributes to muscle protein deposition and bone mineralization. If dietary P is insufficient to meet the animal’s requirements, performance is impaired [63].

In contrast, the FW of the animals fed the DRP + P diet was similar to that of the DB group, indicating that phytase was effective at improving P availability, even under low-P conditions. Thus, P and the excretion of other nutrients may have been reduced, potentially mitigating environmental pollution [64]. These results demonstrate that the enzyme is effective at improving nutrient utilization while suggesting potential environmental benefits. The combined evaluation of 95% prediction intervals and leave-one-out analysis demonstrated high statistical robustness and biological consistency of the performance responses.

The stability observed in sensitivity analyses confirmed the absence of a disproportionate influence from any single study, reinforcing the reliability of the pooled estimates. In contrast, the wide 95% prediction intervals for DWG, DFI, and FC indicate considerable residual heterogeneity, suggesting variation in the magnitude and, potentially, the direction of responses due to multiple sources of variation, including diet formulation, nutritional matrix, ingredient type and source, phytase dose, interactions with vitamin D and calcium, animal age and genetics, duration of supplementation, health status, and management practices. FW exhibited relatively narrower 95% prediction intervals, indicating lower residual heterogeneity and greater predictive consistency, suggesting greater stability as an integrative indicator of phytase effects than more dynamic traits, such as DFI and FC.

### 4.2. Carcass Characteristics

Carcass characteristics of pigs fed diets supplemented with phytase derived from *Buttiauxella* spp. (250, 500, and 1000 FTU/kg) and C. braakii (1000, 2000, and 3000 FYT/kg) were evaluated by Dersjant-Li et al. (2017) and Silva et al. (2019), respectively [14,33]. None of these studies reported negative effects of phytase on carcass traits, which is consistent with the findings of the present meta-analysis. However, compared with the DRP diet, the DB diet reduced this parameter by 1.82 cm^2^. In contrast, Lozano et al. (2011) reported increased muscle depth in finishing pigs fed nutritionally restricted diets supplemented with *A. niger* phytase (500 and 1000 FTU/kg) [16]. Silva et al. (2022) reported an increased lean meat percentage in pigs fed low-P diets supplemented with 4500 FTU/kg of phytase (*Aspergillus oryzae*) [17].

These effects may be attributed to increased insulin sensitivity, which enhances the utilization of energy derived from myo-inositol released by phytase, thereby promoting muscle growth and reducing body fat [65]. Greater phytase responsiveness in males likely reflects their higher lean growth potential and phosphorus demand, enabling more efficient utilization of nutrients released by phytate hydrolysis compared with barrows [55]. However, these effects were not observed in the present study when DRP + P was evaluated, suggesting that dietary composition and nutritional factors should be considered.

Notably, this study was based on a compilation of results from studies conducted in different countries under diverse environmental and production conditions, as well as distinct experimental designs, including variations in diet type, nutritional composition, genetics, nutrient reduction, and phytase inclusion levels. Overall, these findings suggest that phytase does not negatively affect carcass characteristics in growing and finishing pigs. Therefore, these factors may explain the high heterogeneity observed in the meta-analysis.

Sensitivity analyses generally indicated greater structural stability and lower residual heterogeneity for carcass traits. This pattern was most evident for CY, LEA, and LMP, which exhibited relatively narrow 95% prediction intervals and high stability in the leave-one-out analysis. In contrast, CW and, particularly, LMY exhibited wider 95% prediction intervals and greater sensitivity to study exclusion, with Farias (2021) exerting a significant influence on pooled estimates and residual heterogeneity [34]. Nevertheless, most variables exhibited satisfactory structural robustness, suggesting that the observed patterns generally reflect the available evidence, although they should be interpreted with caution because of the limited number of studies.

### 4.3. Economic Impact of Phytase

This meta-analysis is the first to assess the economic impact of phytase in growing and finishing pigs using meta-analytic data in economic modeling. These economic results are consistent with those reported by Oliveira et al. (2010), who reported no effect on carcass characteristics but reported a reduction of US$ 12.58 in production costs with the DRP + P diet compared with the DB diet [66]. Similarly, Dersjant-Li et al. (2018) reported that compared with the DB diet, phytase supplementation reduced feed costs without affecting animal performance, resulting in net profit increases of US$ 3.58 (500 FTU/kg) and US$ 4.21 (1000 FTU/kg), respectively [20].

However, [67] reported no effects of phytase on economic viability, as the enzyme did not affect the cost per unit of weight gain, cost index, or economic efficiency index. Economic outcomes are strongly influenced by market conditions (ingredient and enzyme prices), nutritional strategies (levels of P and other nutrient reduction), phytase source, inclusion level, and stage of supplementation [67].

The mean GM and cost variation differed numerically among the treatments. Under scenarios in which the phytase price increased to 2×, 3×, and 4× the baseline value, the DRP + P diet yielded higher GM than the DB and DRP diets did and showed negative cost variation, indicating superior economic performance and operational efficiency [68], supporting its use in swine production. These economic performance indicators are important for guiding decision-making regarding management and nutritional strategies and ensuring the efficient use of financial resources and production factors to achieve improved economic outcomes [69].

Although phytase supplementation did not significantly affect profit or nutritional cost per ton in this study, the magnitude of the observed mean differences may be economically relevant at the industrial scale of swine production. Compared with DB and DRP, DRP + P increased profit per animal by US$ 2.663 and US$ 5.143, respectively. In terms of nutritional cost per ton, compared with DB, DRP + P reduced costs by US$ 68.911 and by US$ 69.851, respectively, without compromising animal performance or carcass characteristics. These economic benefits were maintained even when the phytase price increased up to fourfold relative to the reference value.

P is generally the third most expensive component in swine diets after energy and protein sources [70]. In this study, soybean meal and dicalcium phosphate were the ingredients with the greatest impact on total diet cost. To achieve better economic performance, DRP and DRP + P followed a nutritional strategy based on reducing digestible P through decreased inclusion of dicalcium phosphate without the application of a phytase matrix.

Given the large scale of production, the substantial capital involved, and the current economic context of feed ingredients, which are subject to constant price fluctuations, even small cost reductions or profit increases can result in significant economic returns [67]. According to USDA data, global pork production in 2025 is 116.7 million tons. Given that the DRP + P diet can reduce costs by US$ 68.911 compared with the DB diet, this represents a potential reduction in feed costs of approximately US$ 8.04 billion worldwide. Given that Brazil is projected to produce 5.7 million tons of pork in 2025, the results of this study represent an approximate reduction of US$ 392.8 million in feed costs.

In the present study, satisfactory results were obtained, suggesting the use of phytase supplementation in growing and finishing pigs. Sensitivity analysis revealed that phytase supplementation remained economically advantageous even at phytase prices up to fourfold higher than the reference value, maintaining profitability and production efficiency.

### 4.4. Study Limitations and Future Directions

This meta-analysis has limitations that should be considered when interpreting the results. With respect to carcass characteristics, only six studies met the inclusion criteria, which limits their statistical power and generalizability. In terms of performance, substantial heterogeneity was observed in the DRP + P vs. DB comparison for the DFI, while high heterogeneity was observed for the DWG, DFI, and FC across all the comparisons. For the carcass traits, high heterogeneity was observed for the CW, BT, and LMY in the DB vs. DRP comparison, whereas in the DRP + P vs. DRP comparison, high heterogeneity was observed only for the LMY. These findings suggest the influence of additional factors on the study outcomes. Egger’s test indicated significant asymmetry for FC, FW, and for CY, LEA, and LMA in some comparisons, suggesting potential publication bias. However, this asymmetry may also reflect between-study heterogeneity, methodological differences, and variability in experimental conditions. Therefore, these findings should be interpreted with caution.

Although leave-one-out analysis indicated high robustness of pooled estimates, the wide 95% prediction intervals observed for DWG, DFI, FC, CW, and particularly LMY indicate substantial residual heterogeneity, limiting response predictability across different experimental contexts. Furthermore, the influence of specific studies, particularly Farias (2021) for CW and LMY, highlights the need for greater experimental standardization and more detailed characterization of sources of variation in future studies [34].

Nevertheless, this study provides valuable insights by systematically synthesizing the available data, identifying factors influencing phytase activity, and revealing consistent patterns that are not evident in individual studies. Future studies should prioritize controlled trials with standardized methodologies and larger sample sizes to improve robustness and external validity. It is recommended to evaluate interactions between phytase, pig sex, and P reduction, as well as to determine optimal phytase inclusion levels and degrees of P reduction in different diet types. In addition, the economic impact of phytase over supplementation should be investigated. Finally, the low identification of moderators in the meta-regression suggests the need to consider dietary factors such as vitamin D, Ca and P levels and sources to better explain the observed heterogeneity.

## 5. Conclusions

In diets with reduced digestible phosphorus, phytase supplementation improves DWG, DFI, FC, and FW in growing–finishing pigs, restoring performance to levels comparable with those of reference diets while reducing feed costs and increasing economic costs. In contrast, DRP compromises productive performance and reduces revenue from nonpremium pigs. Phytase inclusion at levels up to 1000 FTU/kg, associated with a reduction of 0.12 percentage points in digestible phosphorus, resulted in favorable productive responses under the evaluated conditions; however, the results for carcass traits should be interpreted with caution because of the limited number of studies and the residual heterogeneity observed. In this context, the successful commercial application of phytase should carefully consider market conditions, input costs, ingredient availability, sex, production system, and other factors inherent to swine production.

## Figures and Tables

**Table 1:** Summary of phytase supplementation studies in growing and finishing pigs, including sex, diet type, P reduction, dose and phytase source.

Studys	Animals, N°	Sex	Diets ^1^	P Reduction, %	Phytase Levels, FTU/kg	Origin of Phytase
Miller et al. [36]	252	Male	BWS	0.11	0–2000	*Aspergillus oryzae*
Guggenbuhl et al. [37]	36	Male	CSRBO	0.11	0–3000	*Trichoderma reesei*
Dersjant et al. [14]	180	Male	BWSC	0.11	0–1000	*Trichoderma reesei*
Dersjant et al. [20]	279	Male	CSW	0.06	0–1000	*Trichoderma reesei*
Torres- Pitarch et al. [32]	108	Mixed	BWR-DDGSw	0.08	0–500	*Escherichia coli*
Veum et al. [38]	252	Barrows	Sorghum-Canola	0.12	0–800	*Aspergillus niger*
Silva et al. [33]	216	Barrows	Corn–Soybean	0.13	0–3000	*Aspergillus oryzae*
Grela et al. [39]	216	Mixed	BWST	0.10	0–1500	*Aspergillus oryzae*
Lagos et al. [40]	300	Mixed	Corn–Soybean	0.11	0–500	*Escherichia coli*
Farias [34]	60	Barrows	Corn–Soybean	0.15	0–1000	*Aspergillus niger*
Lagos et al. [35]	240	Mixed	Corn–Soybean	0.11	0–500	*Escherichia coli*
Babatunde and adeola [41]	96	Mixed	Corn–Soybean	0.13	0–1000	*Aspergillus oryzae*
Silva et al. [17]	216	Barrows	Corn–Soybean	0.13	0–4500	*Aspergillus oryzae*
Souza et al. [42]	78	Mixed	CSW	0.12	0–500	*Trichoderma reesei*
Williams et al. [43]	540	Male	Corn–Soybean	0.11	0–2000	*Aspergillus oryzae*
Jlali et al. [13]	90	Mixed	CSS	0.12	0–500	*Trichoderma reesei*
Buzek et al. [44]	80	Barrows	Corn–Soybean-Canola	0.10	0–2000	*Escherichia coli*

^1^ Basal ingredients used for diet formulation: BWS: barley–wheat–soybean; CSRBO: corn–soybean–rapeseed–Barley–Oat; BWSC: barley–wheat–soybean–corn; CSW: corn–soybean–wheat; BWR-DDGSw: barley–wheat–rapeseed–DDGSw; BWST: barley-wheat–soybean-triticale; CSS: corn–soybean-sunflower.

**Table 2:** Summary of standardized mean differences for the performance variables included in the meta-analysis and their analyses by different groups.

Groups	N° of Comparisons (k)	Random Effects Model			Heterogeneity		Egger
		WMD	95%CI	*p*	I^2^	*p*	*p*
**Daily weight gain (DWG), kg/day**							
DB × DRP	46	0.063	0.028–0.098	<0.001	89%	<0.001	0.121
DRP + P × DB	46	0.008	−0.010–0.026	0.374	73%	<0.001	0.441
DRP + P × DRP	46	0.068	0.026–0.109	<0.001	93%	<0.001	0.149
**Daily feed intake (DFI)** **, kg/day**							
DB × DRP	46	0.098	0.019–0.177	0.014	74%	<0.001	0.601
DRP + P × DB	46	0.016	−0.024–0.057	0.433	40%	<0.001	0.501
DRP + P × DRP	46	0.106	0.003–0.209	0.043	84%	<0.001	0.939
**Feed conversion (FC)** **, kg/kg**							
DB × DRP	46	−0.077	−0.171–0.017	0.108	88%	<0.001	<0.001
DRP + P × DB	46	−0.030	−0.120–0.060	0.515	91%	<0.001	<0.001
DRP + P ×DRP	46	−0.115	−0.225–−0.005	0.039	93%	<0.001	<0.001
**Final weight (FW), kg**							
DB × DRP	42	4.621	2.138–7.103	<0.001	12%	<0.001	<0.001
DRP + P × DB	42	0.810	−0.088–1.707	0.077	2%	0.001	0.030
DRP + P × DRP	42	4.630	2.422–6.838	<0.001	10%	<0.001	<0.001

BD: basal diet; DRP: diet with reduced P; DRP + P: DRP diet supplemented with phytase.

**Table 3:** Summary of meta-regression analyses of moderators affecting performance variables across different model structures and factor combinations.

Performance		Meta-Regression Model					
Moderators	N° of Comparisons (k)	DB × DRP		DRP + P × DB		DRP + P × DRP	
		*p*	R^2^	*p*	R^2^	*p*	R^2^
**Daily weight gain, kg/day**							
Origin of Phytase	46	-	-	0.286	0%	0.373	1%
Phytase Levels, FTU/kg	46	-	-	0.802	12%	0.853	0%
P reduction	46	0.171	55%	0.785	0%	0.527	4%
Diet	46	0.796	0%	0.299	1%	0.392	0%
Sex	46	0.802	0%	0.142	13%	0.549	10%
**Daily feed intake, kg/day**							
Origin of Phytase	46	-	-	0.806	0%	0.874	0%
Phytase Levels, FTU/kg	46	-	-	0.988	34%	0.795	7%
P reduction	46	0.360	20%	0.978	0%	0.531	6%
Diet	46	0.306	1%	0.101	10%	0.148	7%
Sex	46	0.208	12%	0.039	22%	0.082	19%
**Feed conversion, kg/kg**							
Origin of Phytase	46	-	-	0.954	3%	0.753	0%
Phytase Levels, FTU/kg	46	-	-	0.935	34%	0.529	39%
P reduction	46	0.638	21%	0.008	36%	0.182	0%
Diet	46	0.491	0%	0.407	0%	0.555	0%
Sex	46	0.865	0%	0.083	1%	0.019	17%
**Final weight, kg**							
Origin of Phytase	42	-	-	0.701	0%	0.436	20%
Phytase Levels, FTU/kg	42	-	-	0.905	72%	0.805	0%
P reduction	42	0.001	80%	0.670	0%	0.000	75%
Diet	42	0.781	0%	0.403	0%	0.273	0%
Sex	42	0.815	0%	0.466	0%	0.622	0%

BD: basal diet; DRP: diet with reduced P; DRP + P: DRP diet supplemented with phytase.

**Table 4:** Summary of subgroup analyses for performance variables across different models and factors.

Performance									
Groups	N° of Comparisons (k)	Meta-Regression Model		Subgroups Analysis	Random Effects Model			Heterogeneity	
		*p*	R^2^		WMD	95%CI	*p*	I^2^	*p*
DB × DRP − FW, kg	42	0.001	80%	P reduction					
	16			≤0.11	2.398	−0.927–5.725	0.157	70%	<0.001
	26			≥0.12	6.779	3.250–10.308	<0.001	8%	0.100
DRP + P × DB − DFI, kg/day	46	0.039	22%	Sex					
	7			Barrows	−0.019	−0.079–0.041	0.531	74%	<0.001
	21			Male	0.070	−0.021–0.162	0.135	34%	0.026
	18			Mixed	−0.012	−0.043–0.019	0.465	0%	0.991
DRP + P × DB − FC kg/kg	46	0.008	36%	P reduction					
	20			≤0.11	−0.253	−0.253–0.056	0.212	88%	<0.001
	26			≥0.12	0.026	−0.060–0.114	0.551	93%	<0.001
DRP + P × DRP − FC kg/kg	46	0.019	17%	Sex					
	10			Barrows	0.008	−0.217–0.235	0.939	98%	<0.001
	18			Male	−0.313	−0.543–−0.083	<0.001	92%	<0.001
	18			Mixed	−0.067	−0.163–0.028	0.165	79%	<0.001
DRP + P × DRP − FW, kg	42	0.000	75%	P reduction					
	16			≤0.11	2.634	−1.209–6.477	0.179	76%	<0.001
	26			≥0.12	4.873	3.489–6.257	<0.001	2%	0.588

BD: basal diet; DRP: diet with reduced P; DRP + P: DRP diet supplemented with phytase.

**Table 5:** Summary of standardized mean differences for carcass characteristics included in the meta-analysis and their analyses by different groups.

Groups	N° of Comparisons (k)	Random Effects Model			Heterogeneity		Egger
		WMD	95%CI	*p*	I^2^	*p*	*p*
**Carcass Weight (CW), kg**							
DB × DRP	13	4.480	−1.517–10.478	0.143	80%	<0.001	0.750
DRP + P × DB	15	0.040	−2.157–2.240	0.971	29%	0.038	0.052
DRP + P × DRP	13	3.352	−1.535–8.240	0.178	72%	<0.001	0.207
**Carcass Yield (CY), %**							
DB × DRP	13	0.067	−1.050–1.185	0.906	65%	<0.001	0.742
DRP + P × DB	15	−0.031	−0.537–0.474	0.902	23%	0.131	0.026
DRP + P × DRP	13	−0.022	−0.395–0.351	0.908	0%	0.054	0.348
**Bacon Thickness (BT), mm**							
DB × DRP	13	0.210	−0.866–1.287	0.701	80%	<0.001	0.695
DRP + P × DB	15	−0.295	−1.322–0.732	0.573	68%	<0.001	0.576
DRP + P × DRP	13	−0.090	−0.295–0.115	0.390	0%	0.983	0.960
**Loin Eye Area, (LEA), cm^2^**							
DB × DRP	13	−1.820	−3.176–−0.464	<0.001	21%	0.425	0.001
DRP + P × DB	15	1.084	−0.249–2418	0.110	25%	0.224	0.025
DRP + P × DRP	13	−0.458	−2.225–1.308	0.610	38%	0.016	0.325
**Lean Meat Percentage (LMP), %**							
DB × DRP	13	−0.250	−1.118–0.617	0.617	60%	0.010	0.015
DRP + P × DB	15	0.268	−0.556–1.092	0.523	60%	<0.001	0.014
DRP + P × DRP	13	0.010	−0.314–0.334	0.951	0%	0.997	0.242
**Lean Meat Yield (LMY), kg**							
DB × DRP	11	9.100	−7.325–25.524	0.277	98%	<0.001	0.435
DRP + P × DB	13	0.532	−0.284–1.349	0.201	43%	0.110	0.234
DRP + P × DRP	11	9.242	−6.980–25.464	0.264	98%	<0.001	0.374

BD: basal diet; DRP: diet with reduced P; DRP + P: DRP diet supplemented with phytase.

**Table 6:** Summary of meta-regression analyses of moderators affecting carcass characteristic variables across different model structures and factor combinations.

Carcass Characteristics		Meta-Regression Model					
Moderators	N° of Comparisons (k)	DB × DRP		DRP + P × DB		DRP + P × DRP	
		*p*	R^2^	*p*	R^2^	*p*	R^2^
**Carcass Weight, kg**							
Origin of Phytase	13	-	-	0.287	0%	<0.001	100%
Phytase Levels, FTU/kg	13	-	-	0.005	99%	<0.001	100%
P reduction	13	<0.001	94%	0.956	0%	<0.001	100%
Diet	13	<0.001	0%	0.160	0%	<0.001	0%
Sex	13	<0.001	0%	0.831	0%	<0.001	0%
**Carcass Yield, %**							
Origin of Phytase	13	-	-	0.878	90%	0.002	0%
Phytase Levels, FTU/kg	13	-	-	0.789	0%	0.278	0%
P reduction	13	-	-	0.302	100%	0.594	0%
Diet	13	0.748	0%	0.001	100%	0.609	0%
Sex	13	0.727	0%	0.745	0%	0.677	0%
**Bacon Thickness, mm**							
Origin of Phytase	13	-	-	0.170	0%	0.717	0%
Phytase Levels, FTU/kg	13	-	-	0.216	35%	0.157	0%
P reduction	13	-	-	0.934	0%	0.821	0%
Diet	13	<0.001	0%	0.712	0%	0.681	0%
Sex	13	<0.001	0%	0.539	0%	0.652	0%
**Loin Eye Area, cm^2^**							
Origin of Phytase	13	-	-	0.136	0%	<0.001	100%
Phytase Levels, FTU/kg	13	-	-	0.341	15%	<0.001	61%
P reduction	13	-	-	0.887	0%	<0.001	100%
Diet	13	0.016	92%	0.566	0%	<0.001	100%
Sex	13	0.050	100%	0.257	0%	<0.001	100%
**Lean Meat Percentage, %**							
Origin of Phytase	13	-	-	<0.001	6%	0.749	0%
Phytase Levels, FTU/kg	13	-	-	<0.001	50%	0.753	0%
P reduction	13	-	-	<0.001	0%	0.891	0%
Diet	13	0.526	0%	<0.001	0%	0.561	0%
Sex	13	0.543	0%	<0.001	0%	0.719	0%
**Lean Meat Yield, kg**							
Origin of Phytase	11	-	-	0.736	0%	<0.001	100%
Phytase Levels, FTU/kg	11	-	-	-	-	<0.001	100%
P reduction	11	-	-	0.046	100%	<0.001	100%
Diet	11	<0.001	0%	-	-	<0.001	0%
Sex	11	<0.001	0%	0.894	0%	<0.001	0%

BD: basal diet; DRP: diet with reduced P; DRP + P: DRP diet supplemented with phytase.

**Table 7:** Summary of subgroup analyses for carcass characteristic variables across different models and factors.

Carcass Characteristics									
Groups	N° of Comparisons (k)	Meta-Regression Model		Subgroups Analysis	Random Effects Model			Heterogeneity	
		*p*	R^2^		WMD	95%CI	*p*	I^2^	*p*
DB × DRP − CW, kg	13	<0.001	94%	P reduction					
	7			≤0.11	6.868	−3.981–17.718	0.214	97%	<0.001
	6			≥0.12	1.325	−0.381–3.031	0.128	0%	1.000
	13	<0.001	0%	Diet					
	9			Corn–Soybean	5.768	−2.483–14.021	0.170	88%	<0.001
	4			Alternative	1.291	−0.458–3.042	0.148	0%	0.999
	13	<0.001	0%	Sex					
	7			Barrows	6.868	−3.981–17.718	0.214	97%	<0.001
	3			Male	1.300	−0.460–3.060	0.147	0%	1.000
	3			Mixed	1.710	−5.200–8.620	0.627	0%	0.988
DB × DRP − BT, mm	13	<0.001	0%	Diet					
	9			Corn–Soybean	0.534	−1.281–2.361	0.561	90%	<0.001
	4			Alternative	−0.104	−0.580–0.371	0.666	0%	0.913
	13	<0.001	0%	Sex					
	7			Barrows	0.831	−1.821–3.483	0.539	92%	<0.001
	3			Male	−0.400	−1.330–0.530	0.399	0%	1.000
	3			Mixed	−0.033	−0.265–0.199	0.781	0%	0.991
DB × DRP − LEA, cm^2^	13	0.016	92%	Diet					
	9			Corn–Soybean	−2.533	−4.315–−0.751	0.005	16%	0.822
	4			Alternative	−0.974	−2.196–0.247	0.118	0%	0.972
	13	0.050	100%	Sex					
	7			Barrows	−3.334	−4.284–−2.383	0.000	0%	0.992
	3			Male	−1.300	−3.107–0.507	0.158	0%	1.000
	3			Mixed	−0.483	−2.040–1.073	0.542	0%	0.762
DB × DRP − LMY, kg	13	<0.001	0%	Diet					
	7			Corn–Soybean	14.717	−12.674–42.110	0.292	98%	<0.001
	4			Alternative	0.656	0.054–1.258	0.032	0%	0.861
	11	<0.001	0%	Sex					
	7			Barrows	14.717	−12.674–42.109	0.292	98%	<0.001
	3			Male	0.970	0.039–1.901	0.041	0%	1.000
	1			Mixed	-	-	-	-	-

BD: basal diet; DRP: diet with reduced P; DRP + P: DRP diet supplemented with phytase.

**Table 8:** Summary of subgroup analyses for carcass characteristic variables across different models and factors.

Carcass Characteristics									
Groups	N° of Comparisons (k)	Meta-Regression Model		Subgroups Analysis	Random Effects model			Heterogeneity	
		p	R^2^		WMD	95%CI	p	I^2^	p
DRP + P × DB − CW, kg	15	0.005	99%	Phytase Levels, FTU/kg					
	6			≤1000	1.025	−1.699–3.750	0.460	44%	0.026
	9			≥1001	−0.055	−2.944–2.833	0.970	27%	0.151
DRP + P × DB − CY, %	15			Diet					
	9			Corn–Soybean	0.337	−0.133–0.807	0.160	0%	0.567
	6			Alternative	−0.393	−1.053–0.266	0.242	31%	0.254
DRP + P × DB − LMP, %	15	<0.001	6%	Origin of Phytase					
	6			*A. oryzae*	0.171	−2.173–2.516	0.886	82%	0.004
	3			*T. reesei*	−0.076	−1.003–0.850	0.871	0%	0.848
	6			Other	0.474	−0.674–1.622	0.418	58%	0.043
	15	<0.001	50%	Phytase Levels, FTU/kg					
	6			≤1000	−0.116	−1.655–1.421	0.881	69%	0.012
	9			≥1001	0.162	−0.762–1.086	0.730	60%	0.006
	15	<0.001	0%	P reduction					
	7			≤0.11	0.846	−1.126–2.818	0.400	83%	<0.001
	8			≥0.12	−0.055	−0.569–0.458	0.832	0%	0.970
	15	<0.001	0%	Diet					
	9			Corn–Soybean	0.610	−0.878–2.099	0.421	75%	<0.001
	6			Alternative	−0.051	−0.601–0.499	0.855	0%	0.882
	15	<0.001	0%	Sex					
	9			Barrows	0.544	−0.960–2.048	0.478	77%	<0.001
	3			Male	−0.076	−1.003–0.850	0.871	0%	0.848
	3			Mixed	0.053	−0.664–0.772	0.883	0%	0.957
DRP + P × DB − LMY, kg	15	<0.001	0%	P reduction					
	7			≤0.11	0.363	−0.291–1.017	0.276	0%	0.916
	6			≥0.12	0.775	−0.828–2.378	0.343	74%	0.006

BD: basal diet; DRP: diet with reduced P; DRP + P: DRP diet supplemented with phytase.

**Table 9:** Summary of subgroup analyses for carcass characteristic variables across different models and factors.

Carcass characteristics									
Groups	N° of Comparisons (k)	Meta-Regression Model		Subgroups Analysis	Random Effects Model			Heterogeneity	
		*p*	R^2^		WMD	95%CI	*p*	I^2^	*p*
DRP + P × DRP − CW, kg	13	<0.001	100%	Origin of Phytase					
	6			*A. oryzae*	2.057	1.101–3.014	0.000	0%	0.387
	3			*T. reesei*	2.267	0.506–4.027	0.012	0%	0.935
	4			Other	4.180	−7.830–16.191	0.495	73%	0.000
	13	<0.001	100%	Phytase Levels, FTU/kg					
	5			≤1000	1.990	0.893–3.087	<0.001	0%	0.269
	8			≥1001	3.633	−2.559–9.825	0.250	70%	<0.001
	13	<0.001	100%	P reduction					
	7			≤0.11	5.825	−1.769–13.420	0.132	95%	<0.001
	6			≥0.12	0.723	−3.620–5.067	0.744	21%	0.747
	13	<0.001	0%	Diet					
	9			Corn–Soybean	3.884	−3.071–10.839	0.273	84%	<0.001
	4			Alternative	2.234	0.484–3.984	0.012	0%	0.970
	13	<0.001	0%	Sex					
	7			Barrows	5.825	−1.769–13.420	0.132	95%	<0.001
	3			Male	2.266	0.506–4.027	0.011	0%	0.934
	3			Mixed	−3.075	−9.985–3.834	0.383	0%	0.817
DRP + P × DRP − CY, %	13	0.002	0%	Origin of Phytase					
	6			*A. oryzae*	−0.346	−1.343–0.650	0.495	35%	0.173
	3			*T. reesei*	−0.100	−1.012–0.812	0.830	0%	0.416
	4			Other	1.605	−1.539–4.751	0.317	84%	0.017
DRP + P × DRP − LEA, cm^2^	13	<0.001	100%	Origin of Phytase					
	6			*A. oryzae*	−2.234	−3.310–−1.159	0.000	0%	0.550
	3			*T. reesei*	1.800	−0.007–3.607	0.051	0%	0.800
	4			Other	0.470	−1.025–1.965	0.537	0%	0.712
	13	<0.001	61%	Phytase Levels, FTU/kg					
	5			≤1000	−2.660	−3.824–−1.496	0.000	0%	0.708
	8			≥1001	0.342	−1.081–1.766	0.637	18%	0.416
	13	<0.001	100%	P reduction					
	7			≤0.11	−2.063	−3.149–−0.977	<0.001	0%	0.364
	6			≥0.12	0.951	−0.227–2.130	0.113	0%	0.724
	13	<0.001	100%	Diet					
	9			Corn–Soybean	−2.050	−3.068–−1.031	<0.001	0%	0.580
	4			Alternative	1.149	−0.073–2.371	0.065	0%	0.713
	13	<0.001	100%	Sex					
	7			Barrows	−2.063	−3.150–−0.977	<0.001	0%	0.364
	3			Male	1.800	−0.007–3.607	0.051	0%	0.799
	3			Mixed	0.322	−1.234–1.878	0.685	0%	0.632
DRP + P × DRP − LMY, kg	11	<0.001	100%	Origin of Phytase					
	6			*A. oryzae*	1.089	0.416–1.761	0.001	0%	0.859
	3			*T. reesei*	1.670	0.739–2.600	<0.001	0%	1.000
	2			Other	21.136	−20.53–62.80	0.320	98%	<0.001
	11	<0.001	100%	Phytase Levels, FTU/kg					
	5			≤1000	1.143	0.364–1.922	0.004	0%	0.763
	6			≥1001	11.206	−9.155–31.568	0.280	98%	<0.001
	11	<0.001	100%	P reduction					
	7			≤0.11	14.901	−12.02–41.83	0.278	98%	<0.001
	4			≥0.12	0.762	−0.981–2.506	0.391	71%	0.042
	11	<0.001	0%	Diet					
	7			Corn–Soybean	14.901	−12.02–41.83	0.278	98%	<0.001
	4			Alternative	0.762	−0.981–2.506	0.391	71%	0.042
	11	<0.001	0%	Sex					
	7			Barrows	14.901	−12.02–−41.82	0.278	98%	<0.001
	3			Male	1.670	0.739–2.600	<0.001	0%	1.000
	1			Mixed	-	-	-	-	-

BD: basal diet; DRP: diet with reduced P; DRP + P: DRP diet supplemented with phytase.

**Table 10:** Feed costs and economic indices for growing and finishing pigs supplemented or not supplemented with phytase.

Variables *	Diets						SEM	*p* Value
	BD	DRP	DRP + P	2× P	3× P	4× P		
Revenue RNP, U$	149.348 a	143.632 c	146.970 b	146.970 b	146.970 b	146.970 b	1.876	<0.001
Revenue BF, U$	173.871	164.680	170.723	-	-	-	0.142	0.142
Nutritional cost/head, U$	67.235	63.999	62.195	62.404	62.788	63.130	1.310	0.893
Profit, U$	82.112	79.632	84.775	84.566	84.181	83.839	1.224	0.831
GM, %	54.50	55.20	58.25	58.11	57.85	57,62	0.006	0.416
Food cost, U$/kg	0.803	0.804	0.734	0.736	0.741	0.745	0.010	0.164
Food cost, U$/ton	803.232	804.172	734.321	736.599	741.140	745.176	10.924	0.164
Cost Variation, %	0.000	2.210	−7.924	−7.638	−7.068	−6.562	1.439	0.161

BD: basal diet; DRP: diet with reduced P; DRP + P: diet supplemented with phytase; GM: gross margin. 2× P, 3× P, and 4× P indicate phytase commercial costs that are equivalent to two, three, and four times the baseline price adopted in the present study, respectively. SEM: Standard Error of the Mean. * Values in dollars. Averages followed by different letters in the columns differ (*p* < 0.05) according to Tukey’s test.

## Data Availability

All the performance and carcass characteristics datasets used in this study can be found online in association with their respective publications [3,13,14,17,20,32,33,34,35,36,37,39,40,41,42,43,44].
